# C-955-04. Endothelial Cells in Grafted Skin Substitutes Form Perfused Vessels and Secrete Therapeutic Proteins into Circulation

**DOI:** 10.1093/jbcr/irag033.153

**Published:** 2026-04-06

**Authors:** Dorothy M Supp, Jennifer M Hahn, Kelly A Combs, Yi Cheng, Kewa Gao, Ping Zhou, Heather M Powell, Aijun Wang

**Affiliations:** University of Cincinnati College of Medicine, Cincinnati, OH; University of Cincinnati College of Medicine, Cincinnati, OH; University of Cincinnati College of Medicine, Cincinnati, OH; UC Davis Health, Shriners Children’s - Northern California, Sacramento, CA; UC Davis School of Medicine, Sacramento, CA; UC Davis School of Medicine, Sacramento, CA; The Ohio State University, Columbus, OH; UC Davis School of Medicine, Sacramento, CA

## Abstract

**Introduction:**

Engineered skin substitutes (ESS) containing autologous keratinocytes and fibroblasts were developed to provide permanent wound closure for patients with massive full thickness burns. Previous preclinical studies demonstrated that approaches that accelerate vascularization of ESS were associated with significantly improved engraftment rates. Incorporation of primary human dermal microvascular endothelial cells (HDMECs) into ESS was shown to enable formation of a putative vascular plexus in vitro, which could improve vascularization after grafting. Hypothetically, ESS containing endothelial cells, in addition to fibroblasts and keratinocytes, can serve not just as a prevascularized skin replacement but can also provide a source for secretion of a therapeutic protein into host circulation. The current study compared two endothelial cell sources, HDMECs and cord blood-derived endothelial progenitor cells (EPCs), to determine the optimal cell source for prevascularization of ESS.

**Methods:**

Primary fibroblasts, keratinocytes, and HDMECs were isolated from deidentified human skin and EPCs were isolated from cord blood, with IRB approval. Lentiviral transduction was used to express human coagulation factor VIII (FVIII), the gene mutated in patients with hemophilia A, in EPCs. HDMECs or EPCs were mixed with fibroblasts for seeding of collagen-based scaffolds. These dermal constructs were subsequently seeded with keratinocytes and incubated for 10 days in vitro before grafting to wounds in immunodeficient mice. Mice were euthanized at 2 or 5 weeks post-grafting for analysis; a subset received intravenous fluorescent lectin injection to assess perfusion. Gene expression was analyzed via real time PCR. Human FVIII levels in murine serum were measured by ELISA. Vessels were stained with species-specific antibodies for CD31 and vascularization quantified by image analysis. Statistical analysis via t test was done using SigmaPlot and *p*<.05 considered significant.

**Results:**

HDMECs expressed significantly more FVIII than EPCs in vitro, but FVIII in EPCs was significantly increased after lentiviral FVIII transduction. HDMECs and EPCs both formed vessel-like structures in ESS in vitro that were perfused by 5 weeks post-grafting. Additionally, human FVIII was detected in serum of all grafted mice. Compared with ESS prepared with HDMECs, ESS with EPCs had increased overall vascularization and increased perfusion of human cell-derived vessels, though the differences were not statistically significant.

**Conclusions:**

Both HDMECs and EPCs organize into tube-like structures in ESS in vitro and form perfused vessels after grafting to wounds. Further, both cell types can be used to release a therapeutic protein into host circulation.

**Applicability of Research to Practice:**

The results suggest that ESS prepared with HDMECs or EPCs can provide local wound closure while serving as a vehicle for systemic gene therapy.

**Funding for the study:**

Funded by a grant from the US Dept. of Defense.

